# A review on the deeper understanding of inflammation and infection of the thoracic aorta

**DOI:** 10.1177/17085381211060928

**Published:** 2022-04-25

**Authors:** Kofi Cox, Ritika Dilip Sundaram, Mara Popescu, Kiran Pillai, Muhammed Kermali, Amer Harky

**Affiliations:** 1 Faculty of Medicine, RinggoldID:4915 St George’s Hospital Medical School, University of London, London, UK; 2 School of Medicine, RinggoldID:223860 University of Glasgow, Glasgow, UK; 3 Faculty of Medicine, RinggoldID:405987 King’s College London, London, UK; 4 Department of Cardiothoracic Surgery, RinggoldID:156669 Liverpool Heart and Chest Hospital, Chester, UK

**Keywords:** Aortic aneurysm, Infection, Microbiology

## Abstract

**Objective:**

To review the current literature regarding infection and inflammation of the thoracic aorta and to summarise its aetiologies, pathogenesis and clinical presentation. Additionally, the authors sought to compare diagnostic methods and to analyse the different management options.

**Method:**

A comprehensive electronic search using PubMed, MEDLINE, Scopus and Google Scholar was conducted to find relevant journal articles with key search terms including: ‘aortitis’, ‘thoracic aortic infection’ and ‘surgical management of infected thoracic aortic aneurysms’. Prominent publications from 1995 till present (2021) were analysed to achieve a deeper understanding of thoracic aorta infection and inflammation, and the information was then collated to form this review.

**Results:**

The literature review revealed that infectious causes are more prominent than non-infectious causes, with Gram positive bacteria such as *Staphylococcus*, *Enterococcus* and *Streptococcus* accounting for approximately 60% of the infections. The authors also noted that *Staphylococcus Aureus* was associated with poorer outcomes. Key diagnostic tools include MRI and multi-slice CT imaging, which are useful imaging modalities in defining the extent of the disease thus allowing for planning surgical intervention. Surgical intervention itself is extremely multifaceted and the rarity of the condition means no large-scale comparative research between all the management options exists. Until more large-scale comparative data becomes available to guide treatment, the optimal approach must be decided on a case-by-case basis, considering the benefits and drawback of each treatment option.

**Conclusion:**

A high index of suspicion and a comprehensive history is required to effectively diagnose and manage infection and inflammation of the thoracic aorta. Differentiating between infectious and inflammatory cases is crucial for management planning, as infectious causes typically require antibiotics and surgical intervention. Over the years, the post treatment results have shown significant improvement due to earlier diagnosis, advancement in surgical options and increasingly specific microbial therapy.

## Introduction

Aortitis is a general term that describes aortic inflammation of any cause. The most common aetiologies of aortitis are non-infectious, particularly large vessel vasculitides such as Takayasu and giant cell arteritis. Infectious causes can be attributed to many pathogens including Staphylococci, Enterococci and Streptococci. If undiagnosed, it can lead to the formation of an aneurysm which may rupture, thereby emphasising the importance of early diagnosis and management in improving outcomes. This review aims to present a recent summary of common aetiologies, microbiology, immunology, diagnostic tools and management options for aortitis.

Aortitis is due to infection or inflammation, and usually includes the vasa vasorum.^1^ In the late 1800s, several causes of infected and inflamed aneurysms were established.^2^ Primary infections of the aorta and infected aortic aneurysms are rare, accounting for approximately 2–3% of all aortic aneurysms. These infections are lethal, often requiring prompt surgical intervention, without which the mortality has been reported at 16–44%.^3^ Infectious and non-infectious aetiologies have been studied extensively to determine management options and assess outcomes.

In the last decade, there have been numerous theories explaining the pathology of aortic infections and classifying them. Primary infected aneurysms can emerge from lymphatic spread or direct contact; whereas secondary infected aneurysms occur as a result of septic embolization typically within the vasa vasorum. Understanding whether the source of the infection is intravascular or extravascular is crucial in recognising the infectious agent. Various studies, have classified infections into different types to further understand the pathways and determine management options.

These five types (Table 1) broadly describe how aortitis can arise due to an infection. There are many ways this can occur, such as direct inoculation, or lodging of emboli within the aneurysm itself. Alternatively, lodging of septic emboli in the vasa vasorum could damage the aortic wall, and consequently lead to an aneurysm formation.^3^ Very few cases of non-aneurysmal infectious aortitis have been reported, however, it is difficult to establish a diagnosis as compared to their aneurysmal counterparts due to the nonspecific clinical manifestations.^4^ A case report by Kanemitsu et al. described a patient who presented with fever and abdominal pain, whose CT scan indicated retroperitoneal fibrosis or inflammatory abdominal aortitis. The diagnosis was confirmed using broad range polymerase chain reaction and DNA sequencing, and the authors suggested that this method is useful in rapidly establishing a final diagnosis.^4^ In cases of infectious aortitis attacks in non-aneurysmal aortas, inadequate management can lead to formation of a mycotic aneurysm and subsequent rupture.

**Table 1.** Types of aortic infections.

Types	Description
Mycotic aneurysm	Usually due to endocarditis leading to septic emboli lodging in the vasa vasorum of the aorta (2)
Microbial arteritis	Whereby the arterial wall is weakened due to colonisation of organisms within the vessel wall. This is reported to be the most predominant cause of infected thoracic aortic aneurysms (ITAA) (3)
Infection from pre-existing aneurysm	During bacteraemia (2)
Post-traumatic false aneurysm	This typically occurs in peripheral arteries, or in the thoracic aorta as a result of endovascular stent grafting (2)
Aortic infection	From a contiguous organ with sepsis (2)

Inflammatory aortic aneurysms account for around 10% of aortic aneurysms and are differentiated from atherosclerotic aneurysms by assessing peri-aortic fibrosis and mural thickening. These are non-infectious, with a predominantly unknown aetiology although studies suggest that 50% of cases may be associated with IgG4 related disease and are linked to mediastinal or retroperitoneal fibrosis.^1^ Regarding non-infectious causes, inflammation of the aorta can typically occur as a result of large cell vasculitides such as giant cell arteritis (GCA) and Takayasu’s arteritis. GCA is more common in elderly women and involves the aorta or its branches in 10–18% of cases.^5^ Other causes include systemic lupus erythematosus, rheumatoid arthritis and HLA-B27 associated spondyloarthropathies.^5^

## Aetiology

### Microbiology of infectious aortitis

Clinical outcomes vary according to the microorganism involved in the infection. Any microorganism can cause aortitis, however, some have a proclivity for infecting the aorta. Poor clinical outcomes have been reported in infection caused by *Salmonella* spp. and *Staphylococcus aureus*.^3^ Previously, most cases of aortitis were related to infective endocarditis, in relation to infection by Enterococcus species and Streptococcus pneumonia.^5^.(Table 2)

**Table 2.** Summary of microbiology in infected aneurysms.

Organism	Gram status	Details
*Staphylococcus aureus*	Gram positive	Poor clinical outcome One of the most common causative microorganisms
Streptococci	Gram positive	Related to infective endocarditis (GABHS) (5) Intravenous drug use (1) Intravascular intervention (1)
*Enterococcus*	Gram positive	Related to infective endocarditis (5)
Pneumococcus	Gram positive	Associated with mycotic aneurysms (1)
*Bacillus* cereus	Gram positive	Biofilm production (6) This could infect indwelling medical devices Also related to bacteraemia seeding of the atherosclerotic plaque
*Salmonella*	Gram negative	Make up for over a third of infective abdominal aortitis (3) Poor clinical outcome
*Haemophilus Influenza*	Gram negative	Associated with mycotic aneurysms

Gram positive bacteria such as *Staphylococcus*, *Enterococcus* and *Streptococcus* account for approximately 60% of the infections.^5^ Recent case reports have detailed the involvement of *Bacillus cereus* which can cause serious infection due to its ability to produce biofilms.^6^ This has a significant impact on bacterial survival, whereby bacteria form colonies of microorganisms and are subsequently able to adhere to surfaces and resist antibiotics. This would allow them to adhere to catheters, and potentially cause nosocomial infection.

Mycotic aneurysms were first described by Osler in the 1800s in the context of bacterial endocarditis. The term ‘mycotic’ could be misleading, as it alludes to involvement of fungal pathogens. However, they have been associated with Group A β-haemolytic *streptococci* (GABHS), *pneumococci*, as well as *Haemophilus influenza*.^1^ More recently, they have been linked to intravenous drug use and possible intravascular intervention.^1^

Gram negative bacilli, mainly *Salmonella*, common in infectious abdominal aortitis are reported to make up of approximately 35% of all infected aneurysms.^3^ A retrospective review by Hsu et al. investigating common microorganisms in infected thoracic aortic aneurysms (ITAA) noted that that aortic infections due to nontyphoid *Salmonella* resulted in poor clinical outcomes.^3^ A higher mortality rate was reported here due to subsequent supra-renal aneurysms, and increased likelihood of rupture due to pathogen invasion of the intima.^2,3^

### Immunology of non-infectious aortitis

The immunological processes in aortitis vary based on aetiology, especially when comparing infectious and non-infectious processes. The presence of inflammatory infiltrates in aortic aneurysms increases the likelihood of decay.^7^ Studies have reported that Th1 cytokines such as IFN-γ aid in the development of early atherosclerosis which is a significant risk factor to developing aortitis. Th2 cytokines and chemokines appear in later stages. Although there is involvement of several inflammatory mediators, Th1 is the most critical in accumulation of inflammatory cells in aortic aneurysms.^7^

### Diagnosis

#### Clinical presentation and Laboratory findings

It is vital to establish an early diagnosis with aortitis due to its severe and life-threatening nature. This can be difficult due to the nonspecific signs and symptoms and differing clinical presentations. It should be suspected in patients over the age of 50, with a history of atherosclerosis or aneurysmal disease if they present with thoracic/back pain, nausea, vomiting and fever ^8^. Nonspecific symptoms and results include fever (75%), thoracic and back pain (60%), abdominal pain (20%), leucocytosis, neutrophilia and elevated inflammatory markers.^9,10^ Other findings include a palpable pulsatile tender mass and positive blood culture. Additionally, interleukin-6 can serve as a useful marker in diagnosing GCA.^10^

### Imaging options

Imaging modalities that are useful in diagnosing aortitis when there is a clinical suspicion are CT angiography (CTA), MR angiography (MRA), ultrasound scanning (US) and positron emission tomography (PET) scanning.^11^ Other aspects of diagnosing aortitis are biopsy, histology and immunochemistry.^5^ Differentiating between infectious and non-infectious aortitis is a key feature of the diagnostic process as the incorrect use of corticosteroids or immunosuppressing drugs instead of antimicrobial drugs in the presence of an infected aneurysm is harmful.^12^ Imaging plays a crucial role in this with non-infected aneurysms typically taking on a fusiform shape and involving longer stretches of the aorta.^13^ Infected aneurysms tend to be more saccular in nature and imaging may also show oedema, peri-aortic gas and fat-stranding.^14^ A saccular aneurysm may also indicate a risk or rupture and so its presence should prompt an accelerated diagnostic work-up to enable immediate treatment.^15^ Clinical presentation and blood cultures alone have significantly less diagnostic value in comparison to imaging with regard to differentiating between infected and non-infected aortic aneurysms.^16^

#### CT Imaging

CT scans can image aortic wall thickening and peri-aortic inflammation, which are cardinal signs of aortic infection.^5^ Despite this, mild inflammation and wall oedema may be missed. CT is performed with the administration of iodinated contrast and is particularly helpful in ruling out other pathologies that may have similar presentations to aortitis (e.g. intramural haematoma, aortic dissection).^5^ Limitations of CT include its sensitivity, the need of exposure to radiation and iodinated contrast which may not be administered to all patients.^11^ Compared to MRI, CT imaging can also show arterial calcifications which may be caused by long standing aortitis (e.g.Takayasu arteritis) ^17^. Multidetector CTA offers high resolution and 3D reconstruction which has made it the imaging of choice when evaluating mycotic aneurysms.^1^

#### MRI

MRI provides good resolution images of the aortic wall and lumen with the possibility of 3D reconstructions and is performed with gadolinium contrast. Non-contrast MR angiography can be used alongside contrast imaging to provide complementary information about the lumen, or as an alternative when contrast is unsuitable.^18^ Compared to CT, MRI does not expose patients to ionising radiation which is advantageous in younger age groups (<35 years old). Areas of aortic inflammation may present as wall thickening, oedema or enhancement on MRI. Disadvantages of MRI include the fact that it is not always available and its unsuitability for patients with implanted devices or who are critically unwell.^8^ Recent studies have found 3D-black-blood MRI is an effective and radiation-free alternative to cross sectional CT imaging for the diagnosis of thoracic large vessel vasculitis, making it a useful modality for investigating non-infectious aortitis.^19,20^ Both MRI and multi-slice CT imaging are useful in defining the extent of the disease and for planning surgical intervention.^21,22^

#### PET

A recently emerging technique is combining the use of 18-fluorodeoxyglucose (18F-FDG) PET with CTA or MRA for initial diagnosis of GCA or Takayasu arteritis. 18F-FDG taken up by macrophages and lymphocytes, thus acting as a surrogate marker of increased activity of inflammatory cells.^23^ It is important to differentiate lineal diffuse uptake in the wall that is characteristic for vasculitis from patchy uptake that is more suggestive of atherosclerotic disease.^24^ CT or MRI can then be used in further morphological assessment of the vessel, thus allowing for a more precise localisation of disease in combined imaging.^11^ A systematic review on large vessel vasculitis revealed specificities of 98% and sensitivities of 90% for GCA patients. 18-FDG PET performed well in assessing Takayasu arteritis as well with pooled specificities and sensitivities of 84%. Limitations identified by the meta-analysis include the need to standardise the parameters used to analyse vascular inflammation.^25^ Other small studies of 18F-FDG PET in Takayasu arteritis have shown sensitivity ranges of 60%–92% and specificities of 88%–100%.^26–28^ These have had inconsistent reference standards, so larger clinical studies should be performed for a more relevant comparison.

#### Angiography and Ultrasound

Despite having been the investigation of choice in the past, angiography is invasive and poses the risk of causing the rupture of the aortic wall which can be very fragile when inflamed.^8^ As such, it remains useful only in situations where aortitis or a mycotic aneurysm cannot be excluded otherwise.^11^ Similarly, ultrasound scanning (US) is not a commonly used modality for diagnosing aortitis, but abdominal or transthoracic US can show circumferential thickening of the aortic wall and can be useful in identifying and assessing aneurysms in the ascending thoracic aorta. Transoesophageal echocardiogram is the gold standard investigation for excluding infective endocarditis which is commonly associated with infectious aortitis. While echocardiography measures the lumen diameter, CT and MRI allow for measuring the external aortic diameter which allows for better imaging of aortic wall pathology.^5^

Where there is clinical suspicion of aortitis, we recommend early MRI or CT imaging as the most crucial step in the diagnostic pathway. Whilst the clinical picture of the patient and other previously mentioned facets of the diagnostic work-up are important, imaging is the investigation which allows for the key differentiation between infectious and non-infectious aortitis. Additionally, early imaging enables the planning of life saving treatment to be expedited, thereby mitigating risks such as aneurysm rupture.

### Management

#### Medical management

One of the initial therapy options for non-infectious aortitis is using corticosteroids, generally 0.5–1 mg/kg prednisolone daily for 1–2 years with the possibility of dose reduction 2–3 months after initiation. Nearly 50% patients can relapse during tapering, requiring additional immunosuppression with drugs such as methotrexate.^7,29^ If infectious aortitis is suspected, it should be rapidly diagnosed and requires broad-spectrum IV antibiotics covering the most likely pathogens.^30^ In the case of infected thoracic aortic aneurysms, ampicillin or cephalosporins, which combat *Salmonella* species, should be the first choice antibiotics when a causative agent is not yet identified by a positive blood culture.^31^ If the patient is taken straight to theatre (for example, if they have a ruptured mycotic aneurysm), then perioperative tissue samples should also be taken so the infecting agent can be identified and a more efficacious and narrow spectrum post-operative antibiotic regimen can be implemented.^32^

### Surgical management

Surgical intervention occurs once the infection or inflammation to the thoracic aorta has progressed to a point where an ITAA has formed. Generally, surgery is advised when the aneurysm’s diameter exceeds 55 mm or rapid expansion is observed using short interval CT.^31^ The rarity of ITAA and the clinical variability of the aneurysm means there are no specific guidelines regarding its treatment and the precise nature of the surgical intervention is decided on a case-by-case basis.^33^ There are four main categories of ITAA and whilst their pathogenesis varies depending on their type, the surgical options similar for all types.^34^

#### Open repair options: In situ versus Extra-anatomic reconstruction

Open surgical management involves resecting the aneurysm, thoroughly debriding the infected tissue, and irrigating with saline, to minimise the risk of re-infection. This is followed by aortic reconstruction using in situ graft placement.^35^ A comprehensive statement from the American Heart Association (AHA) regarding the treatment of infected aortic aneurysms outlines five major benefits of in situ aortic reconstruction over extra-anatomic bypass.^36^ The five benefits are:

1. Rifampicin-soaked (and other conduits) are readily available
2. Greater versatility of in situ conduits versus extra-anatomical ones.
3. There are fewer long-term complications (limb amputation, aortic stump rupture, infection recurrence) with an in-situ approach.
4. Lower complication rates in patients with aortic infection secondary to aorto-enteric fistulas.
5. Higher long-term survival rates within situ repair versus extra-anatomic bypass.

It is noteworthy that extra-anatomic bypass is theoretically not possible in the context of an ITAA, however, the benefits of in situ reconstruction are still relevant.^36^ Müller et al. explain that for supra-renal infected aortic aneurysms (i.e. ITAA), the anatomical difficulty and need to revascularize the intercostal, visceral and renal arteries simultaneously means that an in situ repair is the only option, with extra-anatomical repair not being feasible in this setting.^37^ Whilst extra-anatomic bypass has been performed in the thoracic aorta, the only existing examples are in the context of complex aortic stenosis and aortic arch atresia in a paediatric setting.^38,39^ The five benefits above imply that an in situ reconstruction would be preferable even if an extra-anatomic bypass was a feasible alternative. The AHA article states the major disadvantage of in situ reconstruction is that the introduction of the graft, a foreign body, into the infected and potentially purulent vascular tissue may promote re-infection. The necessity of in situ repair in the case of ITAA means that the infected tissue cannot be bypassed and so a pedicle omental flap may be used to bring immune tissue from the omentum to the graft site in an effort to reduce post-operative infection rates.^40^

### Graft materials

#### Antibiotic bound grafts

The opinion of the literature varies regarding what the best graft material is for in situ repair, but there are certainly more popular options. Gupta, Bandyk and Johnson postulated that rifampicin-bonded, gelatin-impregnated antibiotic Dacron grafts were useful in treating staphylococcal infections of the aorta; being especially effective against *S. Aureus*, which is the most common pathogen infecting pre-existing aneurysms.^41,42^ Rifampicin-bound grafts have also been found to be more resistant to post-operative infection than Dacron grafts alone.^43^ Although not directly comparable to treating aortic infection in humans, recent animal studies have concluded that a novel antibiotic combination of minocycline, chlorhexidine and rifampicin has an even lower rate of post-operative infection (25%) versus Dacron alone (75%) or poly-tetra-flour-ethylene (87.5%) in resisting polymicrobial graft infection.^44^ A similar animal pilot study also concluded that grafts bound to these same three antibiotics proved highly effective at reducing graft infection for at least 2 weeks post-operatively which implies that the future of antibiotic bound grafts may be this triple approach rather than rifampicin alone.^45^ However, more studies and human trials are needed to confirm this.

#### Silver-bound grafts

Grafts bound with silver salts have also been shown to be highly effective when used as replacement conduits for the infected thoracic aorta. Batt et al. performed a prospective study where they analysed the use of an InterGard Silver (IGS) collagen and silver acetate–coated polyester graft in replacing mycotic aortic aneurysms (and in redo operations for infected grafts) concluding that secondary infections were less common with the IGS versus the rifampicin-bound graft.^46^ This is supported by Tambe, Sampath and Modak who’s research comparing antibiotics versus antiseptics in medical devices showed that medical devices with silver salts were better at preventing bacterial growth than those containing rifampicin, especially in the case of antibiotic resistance. They concluded that the development of bacterial resistance to antiseptics is negligible, while certain bacteria developed antibiotic resistance far quicker.^47^

#### Cryopreserved allografts

Corvera et al. wrote extensively about the use of cryopreserved allografts in relation to treating thoracic and thoracoabdominal mycotic aneurysms and their work is the largest recorded study of cryopreserved allografts in the thoracic aorta.^48^ They conclude from their treatment of 50 patients from 2006 to 2016 that the use of cryopreserved allografts was associated with good resistance to re-infection owing to the lack of a need to introduce a foreign graft material into infected tissue. They also claim a 64% survival outcome for their patients and identified key drawbacks of cryopreserved allografts: early rupture and pseudoaneurysm formation. The researchers acted on this by ensuring close early observation for suture line disruption or aneurysmal changes to the cryopreserved allograft, however, this early limitation remains the main drawback of this graft approach. McCready et al. also concluded that long-term degeneration of cryopreserved allografts was rare; suggesting that in the absence of initial complications or significant patient comorbidities at the time of operating, they may be a strong candidate for an effective long-term graft type.^49^

#### Endovascular stenting

Endovascular repair for mycotic aortic aneurysms (MAA) was firstly described in 1998, offering a less invasive management method compared to open repair (OR).^50^ Advantages of this method include its adequacy for critically ill patients and avoiding: thoracotomy, aortic cross-clamping, extracorporeal circulation and the manipulation of fragile and inflamed aortic tissue.^11,51^ It can also be used in staged procedures in order to delay open repair until the patient is more stable.^50^ The drawback of endovascular repair is the potential increased rate of graft infection, sepsis, recurrent MAA or fistulation since the stent graft is placed in an infected area with no previous debridement.^51^

A European retrospective multicentre study on MAAs has shown 91% survival at 30 days, 19% fatal infection-related complications (most during the first year post op), 55% survival at 5 years with few serious late infection complications when using endovascular aneurysm repair. Short term mortality rates for OR range from 20% to 40% with significant short- and long-term morbidities from the operation.^52^ A retrospective Swedish nationwide study on mycotic thoracic aortic aneurysms showed that endovascular repair as the predominant management option in Sweden. Survival was 92% at 30 days and 72% at 5 years. It showed comparable frequency of infection-related complications to that reported by Hsu et al. for OR: 17% vs 18%.^53,54^

Stellmes et al. found that endovascular repair to thoracic aortic aneurysm feasible in an emergency setting but noted the importance of life-long clinical monitoring and follow up. Notably, 1/6 of their cases died following endovascular repair and 2/6 of their patients had to undergo second operations where an open repair was performed owing to inadequacies of the former endovascular approach as a permanent solution.^21^ This stance is shared by Ting et al. who conclude that open surgery with in situ aortic reconstruction is associated with favourable outcomes and good long-term results.^55^

Due to the lack of RCTs and head-to-head studies on this relatively rare topic there are no clear guidelines on management of ITAAs and further studies with longer follow ups should be performed to clarify this. Ultimately the management choice should be tailored for each case by considering factors such as the location of the aneurysm, patient comorbidities and the infective pathogen. Regardless of the surgical approach to ITAA repair, post-operative antibiotics play a crucial role in the prevention of re-infection. The timespan for how long antibiotics should be taken post-operatively is very varied but Müller et al., recommended a minimum of 3 months and only to discontinue antibiotics when close examination rules out any chance of remaining infection; based off their own institutional analyses throughout the 1980 and 1990s.^37^

## Outcomes

A 2019 Study evaluated outcomes in patients with non-infectious aortitis following surgical repair of thoracic aortic aneurysms and dissections.^56^ 16 patients with aortitis were followed up over a median of 3.6 years and eight had ‘Significant aortic events’ including further dissections and new aneurysm formation. Low patient numbers mean that the findings of this study may not be consistent with the outcomes experienced by all patients with non-infectious aortitis, however, the scarce literature means this study adds significantly to a generally lacking knowledge base. Prognosis in the short to mid-term is highlighted by a similar case series evaluating 64 patients with non-infectious aortitis (median follow up of 15.4 months) which found a death rate of 9.4%.^57^ A 2014 study of 156 patients who were operated on due to inflammatory diseases of the thoracic aorta provides follow up over a longer time period: they quote a Kaplan–Meier survival rate of 55% at 8 years.^58^ On balance, the scarcity of literature, variability of management and diversity of patient factors makes is hard to predict exactly what outcome a patient with non-infectious aortitis might expect.

Regarding the outcomes of infectious aortitis and mycotic aneurysms, the literature is again lacking and largely focuses on abdominal rather than thoracic aortic pathology. A 2018 paper looking at the outcomes of surgically repaired Mycotic Aortic Aneurysms showed a 5-year survival rate of 71%^54^ with complications including sepsis, graft infection, mycotic aneurysm recurrence and aorto-oesophageal fistula formation. Other studies have quoted similar 5-year survival rates of 71% and 74.9%, respectively.^32,59^ Whilst operative management is associated with risks, untreated infective aortitis has been shown to be associated with a high risk of aortic rupture with a subsequent high mortality.^11^ A systematic review into the management of mycotic aortic aneurysms concluded that EVAR appears to be associated with superior short term survival (without late disadvantages) in comparison to OR.^60^ Of note, the review also concluded that a supra-renal aneurysm location was associated with poorer outcomes, suggesting that thoracic mycotic aortic aneurysms have a worse prognosis in comparison to their abdominal counterparts. Other factors associated with poor prognosis include: advanced patient age, non-salmonella infection/pathogen virulence, non-operative management and the level of infection prior to operating.^61,62^ Overall, the scarcity of literature looking specifically at outcomes in patients with surgically treated thoracic aortic aneurysms and the variability of patient and treatment factors means that the complication rates in any one study may not accurately reflect the risk of developing that complication for the wider patient population.

## Summary

Inflammation and infection of the thoracic aorta are rare and dangerous conditions with a myriad of aetiologies and a reported mortality of 30–50%. Non-infectious causes are primarily accredited to large vessel vasculitides, whereas infectious causes tend to be because of bacterial infections, namely by Gram positive *Staphylococci*, *Enterococci* and *Streptococci* and by Gram negative bacteria such as *Salmonella*. Early diagnosis aids in reducing mortality and is made by clinical examination, laboratory testing and CT, MRI and ultrasound imaging. Regarding the infected thoracic aorta, management typically consists of broad-spectrum antibiotic use and then, provided the patient is a suitable surgical candidate, open or endovascular techniques are used to replace the infected and often aneurysmal aorta. Whilst larger scale research and trials would allow for clearer treatment guidelines to be produced, the complexity of this disease means that management on a case-by-case basis is likely to remain the primary approach for treating patients suffering from infected and inflamed thoracic aortas.

## Conclusion

This review has detailed several avenues of thoracic aortitis and the investigative and management options based on aetiology. Typically, a high index of suspicion and a comprehensive history is required, and, due to the rarity and nonspecific clinical symptoms, there is often an ambiguous diagnosis. Differentiating between infectious and inflammatory cases is crucial for management planning, as infectious causes require antibiotics and surgical intervention. Over the years, the post-treatment results have shown significant improvement due to early diagnosis, advancement in surgical options and increasingly specific microbial therapy.
